# Interactive effects of elevated CO_2_ concentration and combined heat and drought stress on tomato photosynthesis

**DOI:** 10.1186/s12870-020-02457-6

**Published:** 2020-06-07

**Authors:** Rong Zhou, Xiaqing Yu, Junqin Wen, Nikolaj Bjerring Jensen, Thayna Mendanha dos Santos, Zhen Wu, Eva Rosenqvist, Carl-Otto Ottosen

**Affiliations:** 1 Department of Food Science, Aarhus University, Aarhus, Denmark; 2 College of Horticulture, Nanjing Agricultural University, Nanjing, Jiangsu China; 3 Department of Plant and Environmental Sciences, University of Copenhagen, Taastrup, Denmark

**Keywords:** Tomato, Elevated CO_2_ concentration, Combined heat and drought, Recovery, Plant physiology

## Abstract

**Background:**

Extreme weather events are predicted to increase, such as combined heat and drought. The CO_2_ concentration ([CO_2_]) is predicted to approximately double by 2100. We aim to explore how tomato physiology, especially photosynthesis, is affected by combined heat and drought under elevated [CO_2_] (e [CO_2_]).

**Results:**

Two genotypes, ‘OuBei’ (‘OB’, *Solanum lycopersicum*) and ‘LA2093’ (*S. pimpinellifolium*) were grown at a [CO_2_] (atmospheric [CO_2_], 400 ppm) and e [CO_2_] (800 ppm), respectively. The 27-days-old seedlings were treated at 1) a [CO_2_], 2) a [CO_2_] + combined stress, 3) e [CO_2_] and 4) e [CO_2_] + combined stress, followed by recovery. The *P*_N_ (net photosynthetic rate) increased at e [CO_2_] as compared with a [CO_2_] and combined stress inhibited the *P*_N_. Combined stress decreased the F_v_/F_m_ (maximum quantum efficiency of photosystem II) of ‘OB’ at e [CO_2_] and that of ‘LA2093’ in regardless of [CO_2_]. Genotypic difference was observed in the e [CO_2_] effect on the gas exchange, carbohydrate accumulation, pigment content and dry matter accumulation.

**Conclusions:**

Short-term combined stress caused reversible damage on tomato while the e [CO_2_] alleviated the damage on photosynthesis. However, the e [CO_2_] cannot be always assumed have positive effects on plant growth during stress due to increased water consumption. This study provided insights into the physiological effects of e [CO_2_] on tomato growth under combined stress and contributed to tomato breeding and management under climate change.

## Background

The atmospheric CO_2_ concentration (a [CO_2_]) is predicted to be up to 443–541 ppm in 2050 and to be the double of the current concentration by 2100 [1]. The a [CO_2_] has already rapidly peaked 414.8 ppm within the average of May, 2019 (https://scripps.ucsd.edu/programs/keelingcurve/2019/06/04/carbon-dioxide-levels-hit-record-peak-in-may/). The effects of elevated [CO_2_] (e [CO_2_]) on plant were extensive from different aspects. For instance, it is known that e [CO_2_] can increase intercellular [CO_2_] (*C*_i_) and net photosynthetic rate (*P*_N_) and decrease the stomatal conductance (*g*_s_), contributing to the increase of water use efficiency in plants [2, 3]. The e [CO_2_] induced stomatal closure or decreased stomatal density with the generation of reactive oxygen species, ABA receptors and ABA itself as prerequisite in plants [4] and it generally improves salicylic acid biosynthesis but represses jasmonic acid pathway in tomato [5]. Moreover, the e [CO_2_] upregulated 22 genes but downregulated 14 genes mainly playing roles in photosynthesis and development on leaves of sugarcane [6]. The e [CO_2_] can reduce the transcriptional alteration and delay leaf senescence in birch [7].

Co-occurrence of different abiotic stresses lead to complex responses of plants that can not be deduced from single stresses [8]. Among the abiotic stresses, increased temperature and water deficit are the two main constraints to agricultural production that often co-occur in the field [9, 10]. The e [CO_2_] in atmosphere contributes to global warming and induces changes in precipitation patterns, which leads to water scarcity in many areas [11]. Along with the e [CO_2_], global change predicts more frequent abiotic stresses such as heat and drought in future climate, which will complicate the effect of e [CO_2_] on plants [12, 13]. To date, most researches have focused on the response of plants to e [CO_2_] at single abiotic stresses. For instance, Jiang et al. (2016) showed that the e [CO_2_] mitigated the damage of moderate and severe drought stress [12]. The e [CO_2_] could enhance water-use efficientcy and improve plant water relation through decreasing leaf *g*_s_ at drought stress [14, 15]. The thresholds for fraction of transpirable soil water when *P*_N_ and *g*_s_ began to drop were lower in plants at e [CO_2_] than a [CO_2_] along with progressive soil drying, indicating the [CO_2_] hindered gas exchange response to drought [16]. On the other hand, the e [CO_2_] generally alleviated the heat stress damage in terms of plant physiology, such as photosynthesis, hydrogen peroxide (H_2_O_2_) generation and stomatal movement characteristics [13, 17].

However, it is important and meaningful to consider the co-occurrence of heat and drought for future crop breeding and management [18]. The crosstalk between e [CO_2_] and combined drought and heat has been investigated in *Arabidopsis thaliana* [19, 20], *Triticum aestivum* [21, 22], *Brassica napus* [23] and C3 grassland [24]. The stress-mitigating e [CO_2_] effect operates in *Arabidopsis thaliana* by up-regulating the antioxidant defense system and decreasing photorespiration [19] and reducing the negative influence on sugar and amino acid metabolism with less extent of metabolic alteration [20]. Fitzgerald et al. (2016) showed that the e [CO_2_] enhanced wheat yield in semi-arid environments with heat waves [21], while de Oliveira et al. (2013) indicated that the effect on wheat biomass and yield was dependent on temperature [22]. The e [CO_2_] improved plant water relations, contributing to alleviate the adverse effect of combined stress on photosynthetic rate at saturating light [23]. The e [CO_2_] alleviated the negative effects of droughts and heat waves on ecosystem net carbon uptake of C3 grassland [24].

**Fig. 1:**
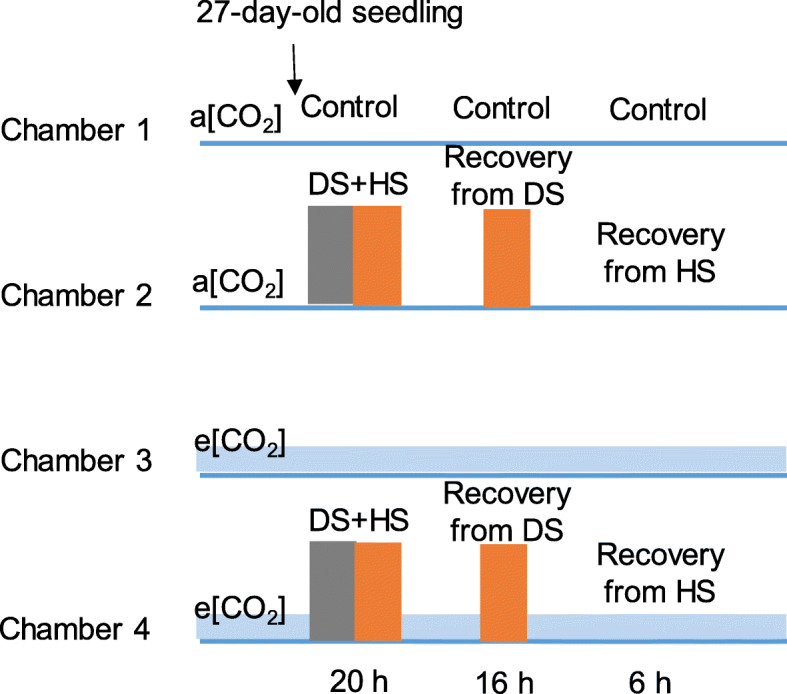
Overview of the work flow. The 27-day-old tomato plants were subjected to the four treatments for 20 h. They included (1) ‘a [CO_2_], 400 ppm [CO_2_] + 25/20 °C + irrigation; (2) a [CO_2_] + combined stress, 400 ppm [CO_2_] + 35/30 °C + no irrigation;(3) e [CO_2_], 800 ppm [CO_2_] + 25/20 °C + irrigation and (4) e [CO_2_] + combined stress, 800 ppm [CO_2_] + 35/30 °C + no irrigation. Then, the plants at combined stress regardless of [CO_2_] were irrigated by water, which was defined as the recovery from DS (drought stress) for 16 h. Finally, the temperature was changed to 25/20 °C, which defined as the recovery from HS (heat stress) lasting 6 h

Tomato is a globally important crop and a model crop for plant research. The e [CO_2_] broadly affects the physiology, metabolism, gene expression and yield of various plants including tomato [3, 25, 26]. These effects induced by the e [CO_2_] have been proven to increase the tolerance of tomatoes to individual heat and drought [13, 27]. However, the effects of e [CO_2_] is not yet fully understood in tomato when being interacted with combined heat and drought. This study aims to is to explore the physiological responses of tomato plants at combined heat and drought to e [CO_2_]. Two genotypes (*S. lycopersicum* and *S. pimpinellifolium*) with different heat susceptibilities were applied to a [CO_2_] + combined stress, e [CO_2_] and e [CO_2_] + combined stress followed by a recovery stage (Fig. 1). Our hypothesis was that (1) the e [CO_2_] alleviate the damage of combined heat and drought stress on tomato; (2) the tomato grown at the e [CO_2_] recover faster than control condition; (3) the effect of CO_2_ concentration differ between genotypes with different heat sensitivities. Our results will provide knowledge on the response of cultivated and wild tomatoes to combined stress in future CO_2_-enriched environmental conditions. This is important to efficiently predict the complex effects of climate changes on crops. We will also help to manage the challenges of future food security under climate changes accompanied by increased population through tomato breeding using tolerant wild species.

## Results

**Fig. 2:**
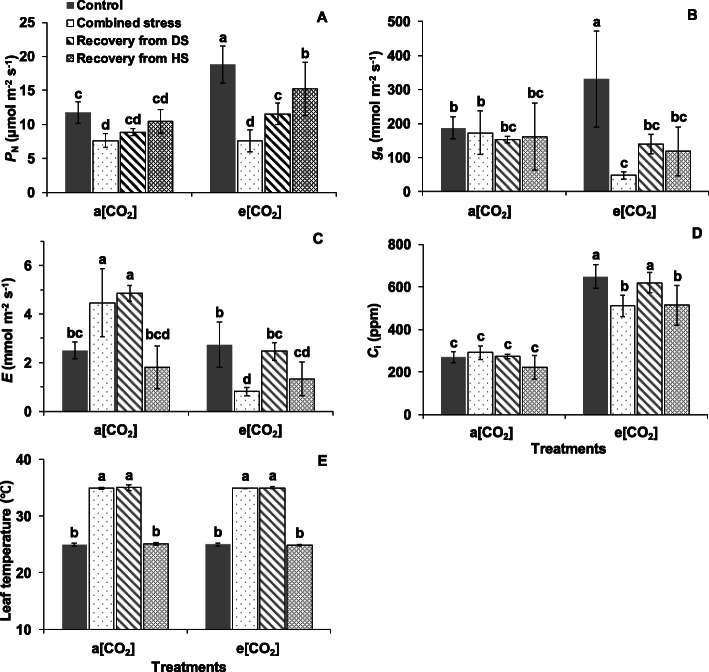
**a** Net photosynthetic rate (*P*_N_), (**b**) stomatal conductance (*g*_s_), (**c**) transpiration rate (**e**), (**d**) intracellular CO_2_ concentration (*C*_i_) and (**e**) leaf temperature in the first fully expanded leaves of tomato ‘OB’ from the top during different treatments. ‘a [CO_2_]’ and ‘e [CO_2_]’ indicates 400 ppm and 800 ppm [CO_2_]. ‘Control’, 25/20 °C; ‘Combined stress’, 35/30 °C (heat stress, HS) + no irrigation (drought stress, DS) for 12 h; ‘Recovery from DS’, 35/30 °C + irrigation for 16 h; ‘Recovery from HS’, 25 °C + irrigation for 4 h. The data represent average values ± SD (*n* = 4). Different small letters showed significant differences (*P* < 0.05)

**Fig. 3:**
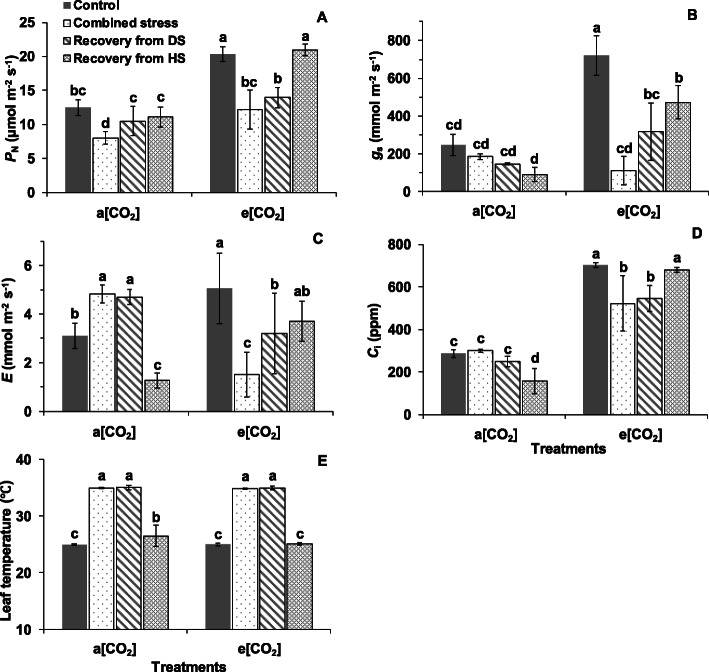
(**A**) Net photosynthetic rate (*P*_N_), (**B**) stomatal conductance (*g*_s_), (**C**) transpiration rate (*E*), (**D**) intracellular CO_2_ concentration (*C*_i_) and (**E**) leaf temperature in the first fully expanded leaves of wild tomato ‘LA2093’ from the top during different treatments. Treatments, sampling and data analysis are as in Fig. 2

The *P*_N_ and *g*_s_ of ‘OB’ and ‘LA2093’ at a [CO_2_] was significantly lower than that at e [CO_2_] at control condition (Figs. 2A, B and 3A, B). Combined heat and drought stress significantly decreased the *P*_N_ of ‘OB’ and ‘LA2093’ plants at both a [CO_2_] and e [CO_2_] (Figs. 2A and 3A). The *P*_N_ of ‘OB’ and ‘LA2093’ plants at a [CO_2_] during the recovery stage showed no significant difference from the controls (Figs. 2A and 3A). The *P*_N_ of ‘OB’ plants at e [CO_2_] during recovery were significantly higher than that during combined stress but lower than control, while that of ‘LA2093’ increased to higher level during recovery from HS (heat stress) and showed no significant difference with control (Figs. 2A and 3A). The *g*_s_ of ‘OB’ and ‘LA2093’ plants at e [CO_2_] during combined stress, and recovery from DS (drought stress) and HS was significantly lower that under control, while that of the plants at a [CO_2_] showed no significant difference (Figs. 2B and 3B). The e [CO_2_] increased the *g*_s_ of the plants from both genotypes as compared with a [CO_2_] (Figs. 2B and 3B). The *E* (transpiration rate) of ‘OB’ and ‘LA2093’ at a [CO_2_] during combined stress and recovery from DS were significantly higher than control (Figs. 2C and 3C). Nevertheless, the *E* and *C*_i_ of ‘OB’ plants at e [CO_2_] during combined stress and recovery from HS and ‘LA2093’ plants at e [CO_2_] during combined stress and recovery from DS were significantly lower than control (Figs. 2C, D and 3C, D). Moreover, leaf temperature of ‘OB’ and ‘LA2093’ at both a [CO_2_] and e [CO_2_] during combined stress and recovery from DS were significantly higher than the respective controls (Figs. 2E and 3E).

**Fig. 4:**
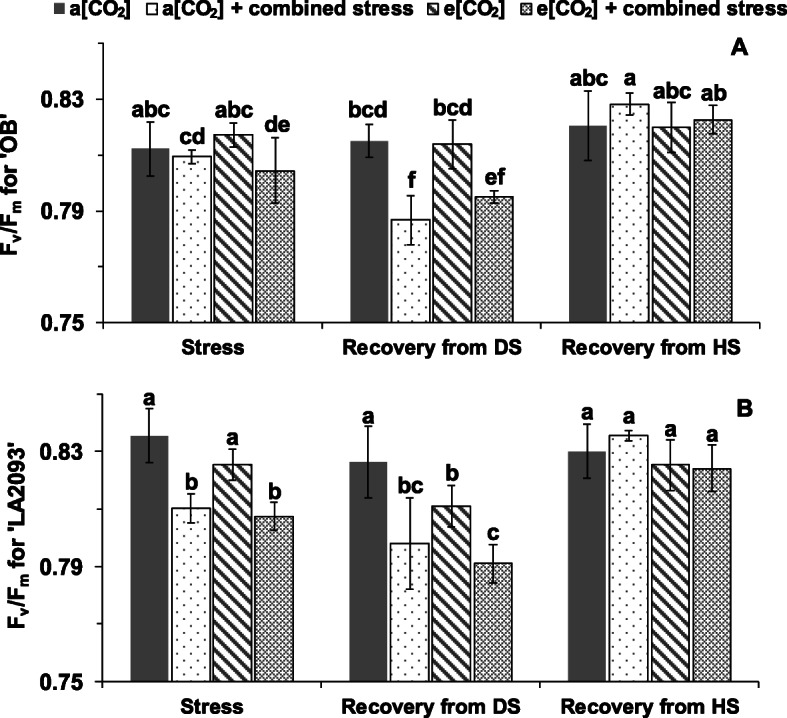
F_v_/F_m_ in the first fully expanded leaves of tomato (**a**) ‘OB’, (**b**) ‘LA2093’ from the top during different treatments. The stage of stress, recovery from DS and recovery from HS lasted 14 h, 18 h and 6 h, respectively. ‘a [CO_2_]’, 400 ppm [CO_2_] + 25/20 °C + irrigation; a [CO_2_] + combined stress’, 400 ppm [CO_2_] + 35/30 °C + no irrigation; ‘e [CO_2_]’, 800 ppm [CO_2_] + 25/20 °C + irrigation; e [CO_2_] + combined stress’, 800 ppm [CO_2_] + 35/30 °C + no irrigation. ‘Recovery from DS’, 35/30 °C + irrigation; ‘Recovery from HS’, 25 °C + irrigation. The data represent average values ± SD (*n* = 4). Different small letters showed significant differences (*P* < 0.05)

The combined stress significantly decreased the F_v_/F_m_ (maximum quantum efficiency of photosystem II) of ‘OB’ at e [CO_2_] and ‘LA2093’ at both a [CO_2_] and e [CO_2_] (Fig. 4). The F_v_/F_m_ of both genotypes during the recovery from DS was significantly lower than the respective controls without stress regardless of CO_2_ concentration (Fig. 4). By comparison, there was no significant difference in F_v_/F_m_ between all the treatments during the recovery from HS (Fig. 4).

**Fig. 5:**
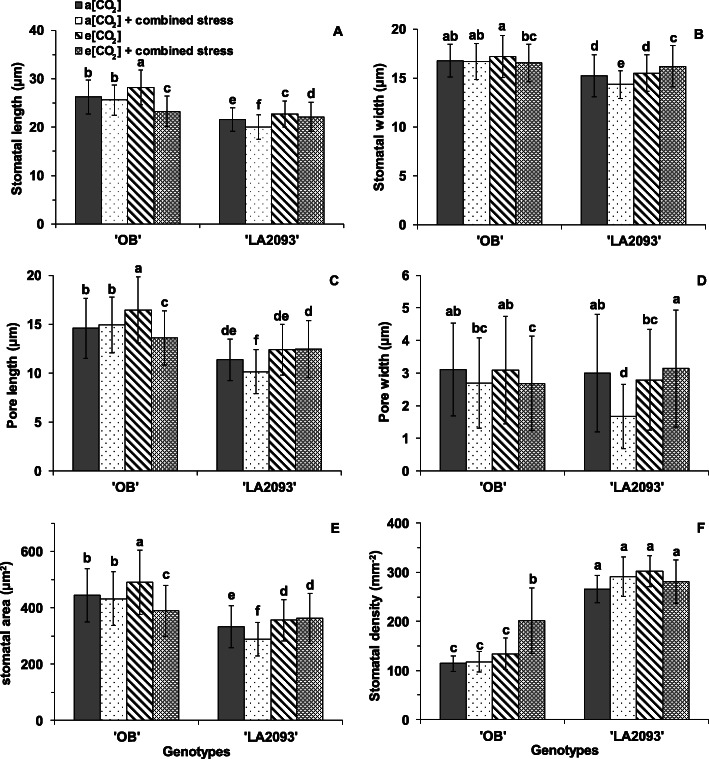
(**a**) Stomatal length, (**b**) stomatal width, (**c**) pore length, (**d**) pore width, (**e**) stomatal area and (**f**) stomatal density in the first fully expanded leaves of tomato ‘OB’ and ‘LA2093’ from the top. ‘a [CO_2_]’, 400 ppm [CO_2_] + 25/20 °C + irrigation; a [CO_2_] + combined stress’, 400 ppm [CO_2_] + 35/30 °C + no irrigation; ‘e [CO_2_]’, 800 ppm [CO_2_] + 25/20 °C + irrigation; e [CO_2_] + combined stress’, 800 ppm [CO_2_] + 35/30 °C + no irrigation. The stress treatments lasted for 20 h. The data represent average values ± SD (*n* = 4). Different small letters showed significant differences (*P* < 0.05)

The stomata and pore length and stomata area of ‘OB’ at e [CO_2_] was higher, while those at e [CO_2_] + combined stress was lower as compared with a [CO_2_] and a [CO_2_] + combined stress (Fig. 5A, C, E). The length and width of stomata and pore as well as stomata area of ‘LA2093’ at a [CO_2_] + combined stress was smallest among the four treatments (Fig. 5A, B, C, D, E). The stomatal density of ‘OB’ at e [CO_2_] + combined stress was highest than the other three treatments (Fig. 5F).

**Fig. 6:**
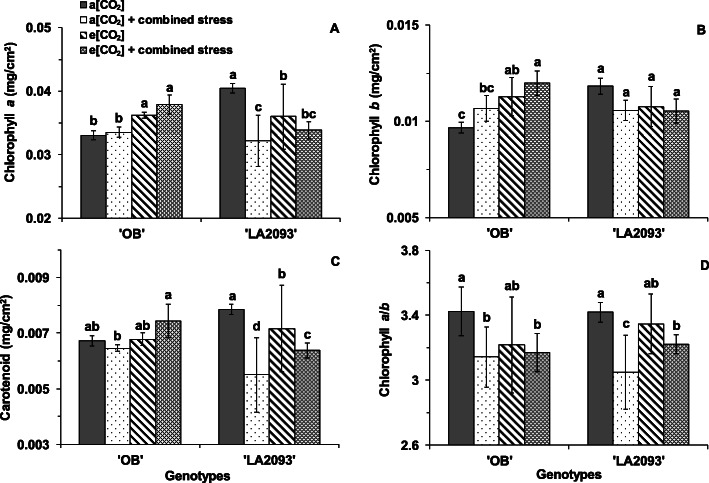
(**a**) Chlorophyll *a*, (**b**) chlorophyll *b*, (**c**) carotenoid and (**d**) chlorophyll *a*/*b* in the first fully expanded leaves of tomato ‘OB’ and ‘LA2093’ from the top. Treatments and sampling are as in Fig. 5. The data represent average values ± SD (*n* = 3). Different small letters showed significant differences (*P* < 0.05)

Combined stress with e [CO_2_] showed distinct effects on the chlorophyll and carotenoid content of the genotypes. The chlorophyll *a* and *b* content in the leaves of ‘OB’ at e [CO_2_] with or without combined stress significantly increased than the control at a [CO_2_] (Fig. 6A, B). However, the chlorophyll *a* and carotenoid content in the leaves of ‘LA2093’ at a [CO_2_] + combined stress, e [CO_2_] and e [CO_2_] + combined stress were significantly lower than control at a [CO_2_] (Fig. 6A, C). The combined stress significantly decreased the chlorophyll *a*/*b* in the leaves of both genotypes regardless of CO_2_ concentration (Fig. 6D).

**Fig. 7:**
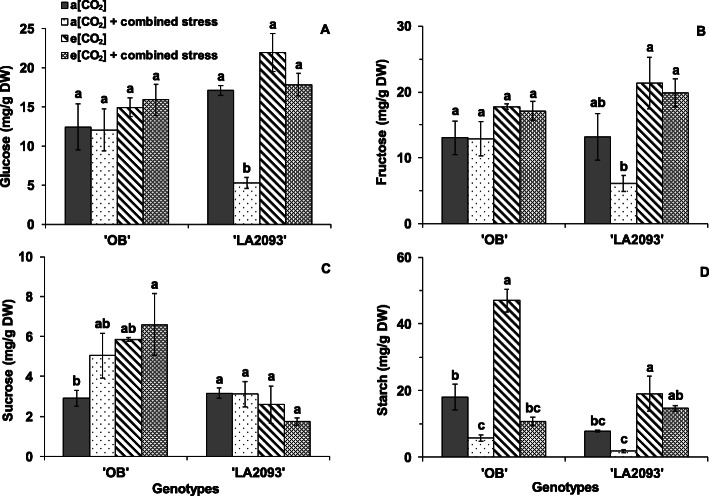
(**a**) Glucose, (**b**) fructose, (**c**) sucrose and (**d**) starch in the first fully expanded leaves of tomato ‘OB’ and ‘LA2093’ from the top. Treatments and sampling are as in Fig. 5. The data represent average values ± SD (*n* = 3). Different small letters showed significant differences (*P* < 0.05)

Similar to the changes in chlorophylls and carotenoid content, the effects of combined stress and e [CO_2_] on carbohydrates content are genotype dependent. The glucose content in the leaves of ‘LA2093’ at a [CO_2_] + combined stress was significantly lower than the other treatments (Fig. 7A). The fructose content in the leaves of ‘LA2093’ at a [CO_2_] + combined stress significantly decreased as compared with e [CO_2_] and e [CO_2_] + combined stress (Fig. 7B). These suggested that the e [CO_2_] had a stress-mitigating effect on the monosaccharide accumulation of ‘LA2093’. The e [CO_2_] + combined stress significantly increased the sucrose content in the leaves of ‘OB’ in comparison with that at a [CO_2_] (Fig. 7C). The starch content in the leaves of ‘OB’ and ‘LA2093’ at e [CO_2_] was significantly higher than that at a [CO_2_] and a [CO_2_] + combined stress (Fig. 7D).

**Fig. 8:**
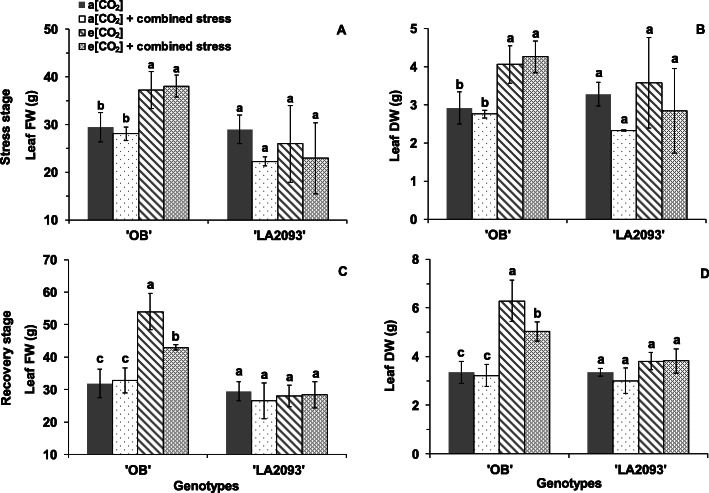
Leaf FW and DW of tomato ‘OB’ and ‘LA2093’ during (**a, b**) stress stage and (**c, d**) recovery stage for 20 h. FW and DW was the abbreviation of fresh and dry weight. Treatments are as in Fig. 5. The data represent average values ± SD (*n* = 3). Different small letters showed significant differences (*P* < 0.05)

**Fig. 9:**
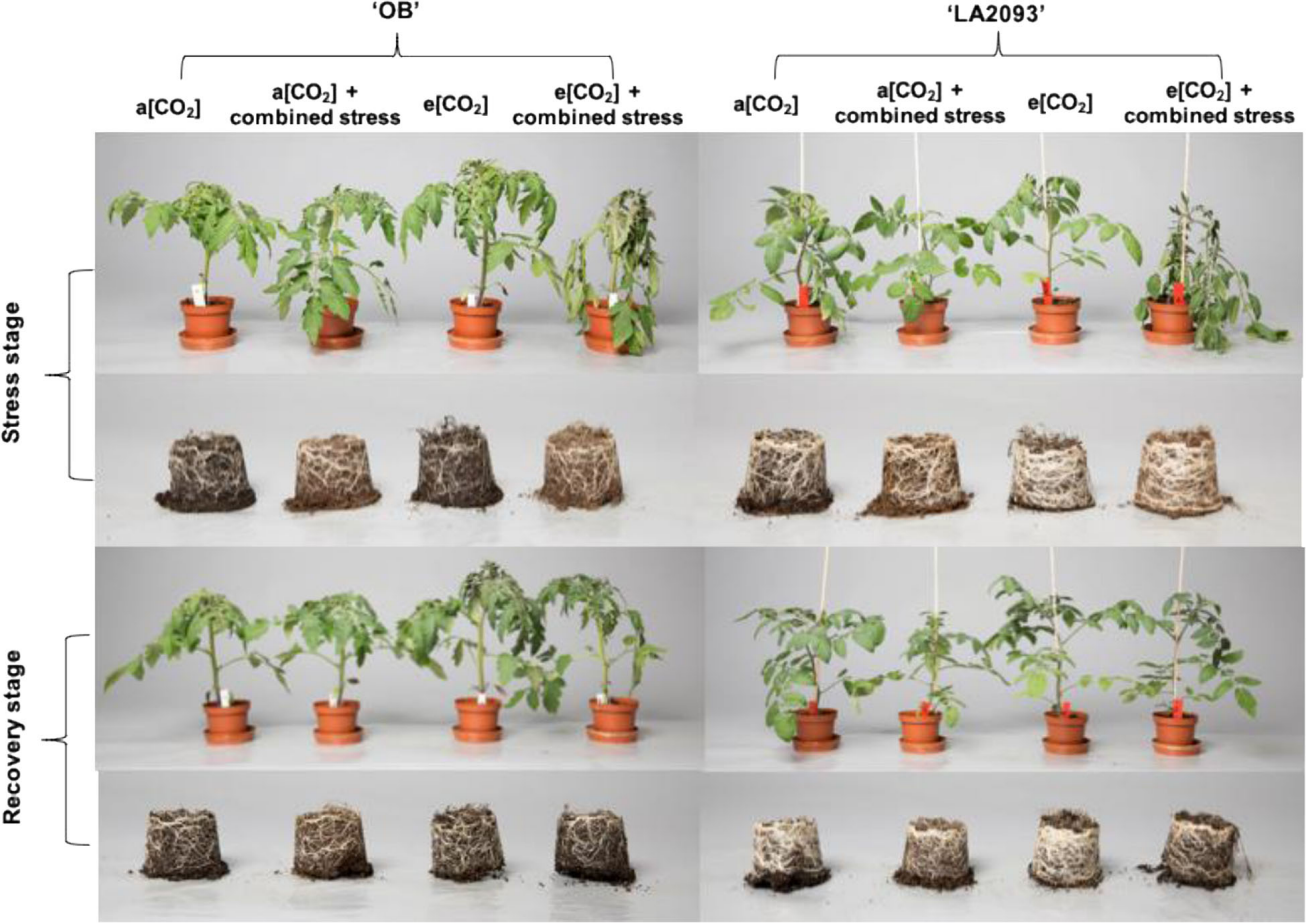
Plant morphology of tomato ‘OB’ and ‘LA2093’ during stress stage and recovery stage for 20 h, respectively. Treatments are as in Fig. 5

The leaf FW (fresh weight) and DW (dry weight) of ‘OB’ at e [CO_2_] were significantly higher than a [CO_2_] regardless of stress after both stress and recovery stage (Fig. 8). Both genotypes grown at e [CO_2_] + combined stress showed wilted leaves and weak growth during the stress; however, the symptoms disappeared after the recovery (Fig. 9). The root did not show visible difference between the four treatments during stress and recovery (Fig. 9).

## Discussion

In recent years, the interaction between e [CO_2_] and abiotic stress conditions has drawn more attention [28]. The predicted increase in [CO_2_] is expected to lead to more frequent extreme high temperature and/or drought episodes [29]. Reduced irrigation together with e [CO_2_] could improve tomato fruit quality at high N supply [27]. The e [CO_2_] enhanced water use efficiency and reduced the negative influence of heat stress on tomato partially due to e [CO_2_]-induced respiratory burst oxidase 1/RBOH1-dependent H_2_O_2_ accumulation and stomatal closure [13]. Our previous results showed that combined heat and drought lead to unique physiological responses of tomatoes as compared with single stress [30]. Thereby, the interaction between the e [CO_2_] and combined stress on tomato physiology is complex, which cannot be determined by the effect of e [CO_2_] and one stress only. However, little is known about whether the e [CO_2_] can change the response of tomatoes at combined stress such as heat and drought.

**Fig. 10:**
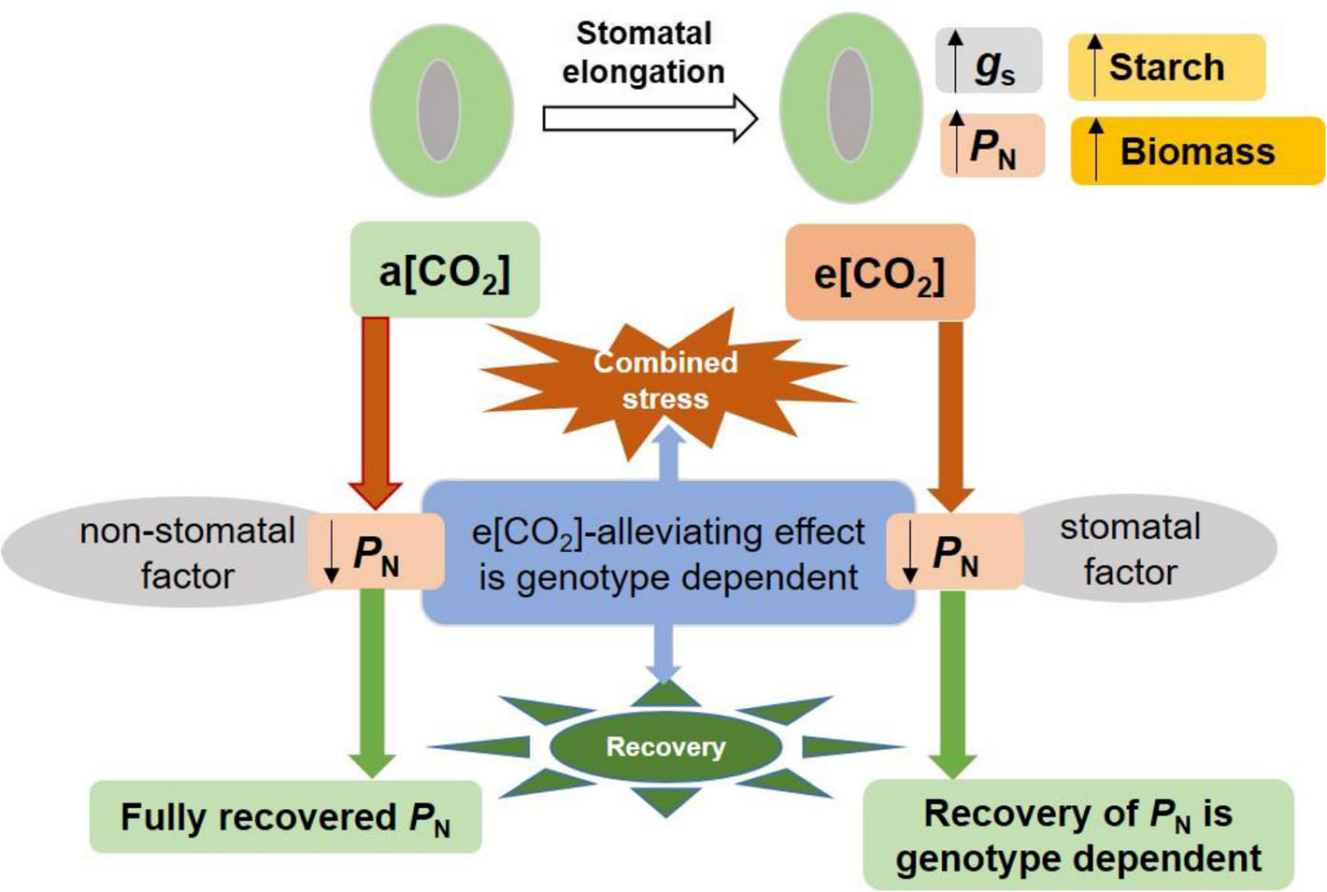
The overall effects of e [CO_2_] on tomato photosynthesis at combined stress and recovery

The physiological and transcriptional response in *Arabidopsis thaliana* showed that photosynthesis, as a fundamental biological process, was a major target of climate extreme like combined heat waves and drought [19]. The e [CO_2_] increased the *P*_N_ of both genotypes in comparison with a [CO_2_] without combined stress, in agreement with the previous studies showing that the plants grown under e [CO_2_] have higher photosynthetic capacity [17, 25]. Zinta et al. (2014) showed that the combined stress inhibited the *P*_N_ of *Arabidopsis thaliana* regardless of [CO_2_] [19]. The lower *P*_N_ of tomato at a [CO_2_] is due to non-stomatal factor since the *g*_s_ and *C*_i_ were unaffected, while the *P*_N_ of tomato at e [CO_2_] accompanied by drops in both *g*_s_ and *C*_i_ was attributed to stomatal factor during combined stress (Fig. 10), according to the description of Von Caemmerer and Farquhar (1981) [31]. Surprisingly, the e [CO_2_] increased the *g*_s_ of both tomato genotypes as compared with a [CO_2_] as the *g*_s_ has been commonly reported to decrease when plants were grown at e [CO_2_] [13, 32]. The increased stomatal area due to the elongation of stomatal as indicated by longer length and unchanged width of stomatal and pore in plants at e [CO_2_] corresponded to the result of *g*_s_ (Fig. 10). By comparison, Chavan et al. (2019) found no difference in the *g*_s_ of wheat plants grown and measured at a [CO_2_] and e [CO_2_, 17], while Zinta et al. (2014) reported the *g*_s_ of *Arabidopsis thaliana* plants grown at e [CO_2_] increased at unstressed condition [19]. However, the reason behind for the inconsistent change of *g*_s_ caused by e [CO_2_] need to be further clarified, which could depend on plant species or developmental stage and be linked to limitation in water or nutrients. Moreover, the e [CO_2_] alleviated the reduction in *P*_N_ in heat-tolerant tomato under combined stress since the *P*_N_ at e [CO_2_] + combined stress was higher than a [CO_2_] + combined stress for ‘LA2093’. This e [CO_2_]-alleviating effect on plant grown at combined heat and drought has been reported in *Arabidopsis thaliana* [19, 20], *Triticum aestivum* [21] and C3 grassland [24]. We suggest that one of the key factors that influenced the alleviation of e [CO_2_] is the sensitivity of the genotype to stress conditions (Fig. 10). More importantly, the *P*_N_ of the heat-tolerant tomato plant at e [CO_2_] fully recovered from the combined stress, while that of ‘OB’ did not, although the *P*_N_ of both genotypes at a [CO_2_] fully recovered. Thereby, the differential genotypic response to the e [CO_2_] effect on the *P*_N_ of plants happened both during stress and recovery (Fig. 10). The underlying genotypic difference need to verify in further studies, particularly for crop improvement to adapt to future e [CO_2_] atmosphere.

The F_v_/F_m_ has been widely and successfully applied to identify stress damage on plants’ photosynthetic apparatus [33]. The e [CO_2_] did not alleviate the negative effects caused by short-term combined stress (12 h) as indicated by the decreased F_v_/F_m_ in both genotypes at e [CO_2_] + combined stress. Accordingly, the alleviating effect of e [CO_2_] on F_v_/F_m_ was observed in plants exposed to a longer duration (7 days) to combined stress compared with 2 day and 4 days of exposure as shown by Zinta et al. (2014) [19]. The tomato plants did not fully recover and continued to deteriorate after watering, as indicated by the consistent low F_v_/F_m_. This result could be explained by the longer duration of heat stress, as the high temperature persisted even though the plants were irrigated. However, the plants fully recovered in terms of F_v_/F_m_ after being exposed to control temperature.

Zinta et al. (2014) showed there were no difference in chlorophylls content of *Arabidopsis thaliana* induced by e [CO_2_] or combined stress for 4 days, but combined stress for 8 days decreased the chlorophylls content regardless of [CO_2_] [19]. The chlorophyll *a* and *b* content of ‘OB’ at e [CO_2_] regardless of combined stress was higher than a [CO_2_] and combined stress decreased the content of chlorophyll *a* of ‘LA2093’ indicating that content depended on genotypes. The chlorophyll *a*/*b* of both genotypes at combined stress decreased in regardless of [CO_2_], indicating an oxidative stress damage [34].

In accordance with Li et al. (2013) [35] and Zinta et al. (2018) [20], the e [CO_2_] enhanced the starch accumulation of plants without stress conditions. The glucose, fructose and starch of ‘LA2093’ at e [CO_2_] + combined stress was higher than that at a [CO_2_] + combined stress. Together with the higher sucrose content at e [CO_2_] + combined stress than a [CO_2_], it could indicate that e [CO_2_] could alter the carbohydrate metabolism and induce carbohydrates accumulation by enhancing photosynthesis without/with combined stress [19, 35].

The leaf FW and DW of ‘OB’ treated at e [CO_2_] and e [CO_2_] + combined stress was higher than that at a [CO_2_] and a [CO_2_] + combined stress during both stress and recovery (Fig. 8), but there were no difference in stem FW and DW (Data not shown). Our finding corresponds to the results by Pazzagli et al. (2016) [14], Chavan et al. (2019) [17], Fitzgerald et al. (2016) [21] and Ainsworth and Long (2005) [32], showing that the e [CO_2_] enhance the biomass production of plants. This could be partially explained by increased photo-assimilates in source leaves of tomato grown at e [CO_2_] as a consequence of increased *P*_N_. In addition, the response of biomass accumulation to e [CO_2_] differed between the tomato genotypes. The ‘LA2093’ showed no significant difference in biomass at e [CO_2_], which could be due to increased leaf dark respiratory rate accompanied by high carbohydrates availability to increase glycolysis and tricarboxylic acid (TCA) cycle flux [35].

Both genotypes grown at e [CO_2_] were more sensitive to combined stress than those grown at a [CO_2_] (Fig. 9). This correspond to the fact that combined stress lead to a higher drop in *P*_N_ of the plants grown at e [CO_2_] than those grown at a [CO_2_]. The explanations could be that the plants at e [CO_2_] generally had more biomass accumulation and higher *g*_s_ as compared with a [CO_2_], leading to higher transpiration and more irrigation requirement. This created a situation that the water depletion of tomatoes at e [CO_2_] happened faster and the growing media become drier [36] and thereby the severity of water deficit at combined stress was higher. The *g*_s_ at drought of *Fagus sylvatica* L. grown from seed for two seasons at e [CO_2_] was higher instead of lower than a [CO_2_], causing faster soil drying [37]. Moreover, the *g*_s_ was less sensitive to induce ABA accumulation in tomato at e [CO_2_] and thereby ABA signialing could not play role in stomatal closure at water deficit condition [36]. There may be harmful influences of the e [CO_2_] for some species on plant-water relations in some cases [38]. Hence, the e [CO_2_] cannot be always assumed have positive effects on plant growth due to other limiting factors.

## Conclusion

The climate changes including e [CO_2_], heat and drought interact with each other under field conditions, which can lead to effects that are remarkably different from single stress exposures. As compared with a [CO_2_], the e [CO_2_] increased the *P*_N_ of both tomato genotypes. However, the combined stress inhibited the *P*_N_ regardless of [CO_2_] and genotype. The F_v_/F_m_ remained lower during recovery from drought, but fully recovered after being exposed to control temperature. The e [CO_2_] environment could enhance the biomass production of plant, but the magnitude of the effects depended on plant genotypes. Genotypic difference was observed in e [CO_2_] effect on the *P*_N_, carbohydrate accumulation, pigment content and dry matter accumulation. The e [CO_2_] cannot be always assumed have positive effects on plant growth during stress conditions due to its deleterious effects on plant-water relations. This study offers insights into the understanding of physiological responses of tomatoes to the interactive effects of future climate factors, which is vital to identify resilient crops for future climate regimes.

## Methods

### Growth conditions

The seeds from tomato ‘OuBei’ (‘OB’) (*Solanum lycopersicum*) were acquired from Northern Nuohua co. LTD, China. The seeds of wild heat-tolerant tomato ‘LA2093’ (*S. pimpinellifolium*) [39] were provided by Nanjing Agricultural University. The seeds were sown in plastic pots (9 cm height, 11 cm-diameter) in two separate compartments (compartment 1: 400 ppm [CO_2_]; compartment 2: 800 ppm [CO_2_]) of a greenhouse at the Department of Food Science, Aarhus University, Aarslev, DK (55.30 N, 10.44E). There were 36 plants per genotype per compartment. The commercial substrate used was Pindstrup 2 (Pindstrup Mosebrug A/S, Ryomgaard, Denmark). The seedlings were grown at approximately 23/16 °C (day/night), relative humidity of 40–60% and 150–300 μmol m^− 2^ s^− 1^ photosynthetic photon flux density (PPFD). The nutrition solution (pH = 6, EC = 2.18, K = 275 ppm, *N* = 191 ppm and *P* = 35 ppm) was applied to 18-day-old seedlings every day. Six days later, the 24-day-old seedlings in compartment 1 were moved to climate chamber 1 with the level of [CO_2_] (400 ppm), while those in compartment 2 were moved to climate chamber 2 with the level of [CO_2_] (800 ppm). The temperature was set at 25 °C for 15 h during daytime (05:00–20:00) and at 20 °C for 9 h during nighttime (20:00–05:00) in both chambers. The setting of relative humidity and PPFD in both chambers was 60% and 300 μmol m^− 2^ s^− 1^ PPFD provided by LED (FL300 sunlight, Fionia Lighting, Søndersø Denmark), respectively. The nutrition solution was applied every day by flooding the bench for about 10 min.

The plants were acclimated in the chambers for 3 days and then the 27-day-old plants in chamber 1 and 2 were equally distributed to chamber 3 and chamber 4, respectively. The four treatments were imposed: (1) a [CO_2_] in chamber 1, 400 ppm [CO_2_] + 25/20 °C + irrigation; (2) e [CO_2_] in chamber 2, 800 ppm [CO_2_] + 25/20 °C + irrigation; (3) a [CO_2_] + combined stress in chamber 3, 400 ppm [CO_2_] + 35/30 °C + no irrigation and (4) e [CO_2_] + combined stress in chamber 4, 800 ppm [CO_2_] + 35/30 °C + no irrigation. There were 18 plants per treatment per genotype. Based on our preliminary test, the irrigation was stopped 4 hours before the high temperature since it took time to make drought stress happen after withdrawing the irrigation, while heat stress happen quickly (10–20 min) after increasing the temperature. Thereby, the irrigation was withdrawn at 16:00 and the temperature was increased to 30 °C at 20:00 for the plants at combined stressed. The combined stress was regarded to begin at 20:00 on day 0. After 20 h of the treatments when the plants at combined stress has shown macroscopic stress phenotype, the irrigation was restarted at 16:00 on day 1 in the same way as the control. This stage from 16:00 on day 1 until 8:00 on day 2 (16 h) was defined as the recovery from DS. The temperature was changed to 25/20 °C from 8:00 until 14:00 on day 2 (6 h) for the plants at combined stress. This stage was defined as the recovery from HS. The experimental time line of treatments was shown in Fig. 1.

### Gas exchange

The *P*_N_, *g*_s_, *E*, *C*_i_ and leaf temperature were measured in the first fully expanded leaf from the top of the plants with four replications. The measurements were taken at 8:00 on day 1 after 12 h of the stress treatments, at 8:00 on day 2 after 16 h of recovery from DS and 12:00 on day 2 after 4 h of recovery from HS using portable photosynthesis system (CIRAS-2, PP Systems, Amesbury, USA). The light level, temperature and CO_2_ concentration settings of the cuvette during the measurements corresponded to the respective growing conditions. The records were taken when the parameters were considered stable. The average of the six records within the last 1 min were regarded as the results.

### Maximum quantum efficiency of photosystem II (F_v_/F_m_)

The F_v_/F_m_ in the first fully expanded leaf from the top of the plants was measured using Handy PEA (Hansatech Instrument, King’s Lynn, England). The leaf was dark adapted for 20 min with a leaf clip. The measurements with four replications were taken at 10:00 on day 1 after 14 h of the stress treatments, at 10:00 on day 2 after 18 h of recovery from DS and 14:00 on day 2 after 6 h of recovery from HS.

### Stomatal traits

Original imprints were obtained by evenly painting the impression material (elite HD+, Zhermack, Badia Polesine, Italy) to the leaf surface. The imprints were transferred by applying nail varnish to the original imprints. Image data was acquired using a Nikon AZ100 microscope (Nikon Corp., Tokyo, Japan) equipped with a Nikon DS-Fi1 camera. There were four imprints from the abaxial leaf surface of the four plants per treatment. For each imprint, four photos were taken at different locations in the slide. Stomata number was counted for each picture excluding those cut by the picture border and stomatal density was calculated per area. For all stomata, stomatal length, stomatal width, pore length, pore width and stomata area (the area of the rectangle encasing the stomata) were investigated.

### Chlorophyll and carbohydrate content

The first fully expanded leaf from the top were harvested during the stress treatments for 20 h. One leaf disk (2.96 cm^2^) per plant were punched using a cork-borer for chlorophyll content measurements with three replications per treatment. The leaf samples were freeze-dried and grounded before carbohydrate content measurements. Chlorophyll and carbohydrate content were determined according to Zhou et al. (2015) [39].

### Fresh and dry weight (FW and DW) of leaf

The leaf FW and DW of the plants with three replications during the stress and recovery was determined using an analytical balance. The leaf DW were measured after putting the fresh samples at 80 °C in a constant flux oven for 48 h.

### Data analysis

The photosynthesis and F_v_/F_m_ data of each cultivar at a [CO_2_] and e [CO_2_] during the control condition, combined stress, recovery from DS and recovery from HS were analyzed by one-way Analysis of variance (ANOVA) (Duncan’s post hoc test) using SPSS 16.0 (SPSS Inc. Chicago, IL). The stomatal, pigment and carbohydrate content as well as biomass data of each cultivars at a [CO_2_] and a [CO_2_] + combined stress, e [CO_2_] and e [CO_2_] + combined stress were analyzed by one-way ANOVA (Duncan’s post hoc test). To perform the one-way ANOVA in SPSS, choose ‘Analyze’ → ‘General Linear Model’ → ‘Univariate’ after inputting data based on the methods from DeCoster and Claypool (2004) [40]. The significant levels were at 0.05.

## Data Availability

The datasets during or analyzed during the current study available from the corresponding authors on reasonable request.

## Abbreviations

a [CO_2_]: atmospheric CO_2_ concentration
ANOVA: Analysis of variance
*C*_i_: intercellular CO_2_ concentration
[CO_2_]: CO_2_ concentration
DW: Dry weight
*E*: Transpiration rate
e [CO_2_]: elevated CO_2_ concentration
F_v_/F_m_: Maximum quantum efficiency of photosystem II
FW: Fresh weight
*g*_s_: stomatal conductance
H_2_O_2_: Hydrogen peroxide
*P*_N_: Net photosynthetic rate
PPFD: Photosynthetic photon flux density
TCA: Tricarboxylic acid

## References

[1] Lemonnier P, Ainsworth EA (2018). Crop responses to rising atmospheric [CO_2_] and global climate change. Food security and climate change.

[2] Ainsworth EA, Rogers A (2007). The response of photosynthesis and stomatal conductance to rising [CO_2_]: mechanisms and environmental interactions. Plant Cell Environ.

[3] Shi K, Li X, Zhang H, Zhang G, Liu Y, Zhou Y (2015). Guard cell hydrogen peroxide and nitric oxide mediate elevated CO_2_-induced stomatal movement in tomato. New Phytol.

[4] Chater C, Peng K, Movahedi M, Dunn JA, Walker HJ, Liang YK (2015). Elevated CO_2_-induced responses in stomata require ABA and ABA signaling. Curr Biol.

[5] Zhang S, Li X, Sun Z, Shao S, Hu L, Ye M (2015). Antagonism between phytohormone signalling underlies the variation in disease susceptibility of tomato plants under elevated CO_2_. J Exp Bot.

[6] De Souza AP, Gaspar M, Da Silva EA, Ulian EC, Waclawovsky AJ, Nishiyama MY (2008). Elevated CO_2_ increases photosynthesis, biomass and productivity, and modifies gene expression in sugarcane. Plant Cell Environ.

[7] Kontunensoppela S, Riikonen J, Ruhanen H, Brosché M, Somervuo P, Peltonen P (2010). Differential gene expression in senescing leaves of two silver birch genotypes in response to elevated CO_2_ and tropospheric ozone. Plant Cell Environ.

[8] Prasch CM, Sonnewald U (2013). Simultaneous application of heat, drought, and virus to *Arabidopsis* plants reveals significant shifts in signaling networks. Plant Physiol.

[9] Mittler R (2006). Abiotic stress, the field environment and stress combination. Trends Plant Sci.

[10] Roy SJ, Tucker EJ, Tester M (2011). Genetic analysis of abiotic stress tolerance in crops. Curr Opin Plant Biol.

[11] Wang YS, Liu FL, Andersen MN, Jensen CR (2010). Improved plant nitrogen nutrition contributes to higher water use efficiency in tomatoes under alternate partial root-zone irrigation. Funct Plant Biol.

[12] Jiang Y, Xu Z, Zhou G, Tao L (2016). Elevated CO_2_ can modify the response to a water status gradient in a steppe grass: from cell organelles to photosynthetic capacity to plant growth. BMC Plant Biol.

[13] Zhang H, Pan C, Gu S, Ma Q, Zhang Y, Li X, Shi K (2019). Stomatal movements are involved in elevated CO_2_-mitigated high temperature stress in tomato. Physiol Plant.

[14] Pazzagli PT, Weiner J, Liu F (2016). Effects of CO_2_ elevation and irrigation regimes on leaf gas exchange, plant water relations, and water use efficiency of two tomato cultivars. Agric Water Manag.

[15] Wullschleger SD, Tschaplinski TJ, Norby RJ (2002). Plant water relations at elevated CO_2_-implications for water-limited environments. Plant Cell Environ.

[16] Liu J, Hu T, Fang L, Peng X, Liu F (2019). CO_2_ elevation modulates the response of leaf gas exchange to progressive soil drying in tomato plants. Agric For Meteorol.

[17] Chavan SG, Duursma RA, Tausz M, Ghannoum O (2019). Elevated CO_2_ alleviates the negative impact of heat stress on wheat physiology but not on grain yield. J Exp Bot.

[18] Jin Z, Zhuang Q, Wang J, Archontoulis SV, Zobel Z, Kotamarthi VR (2017). The combined and separate impacts of climate extremes on the current and future US rainfed maize and soybean production under elevated CO_2_. Glob Chang Biol.

[19] Zinta G, AbdElgawad H, Domagalska MA, Vergauwen L, Knapen D, Nijs I (2014). Physiological, biochemical, and genome-wide transcriptional analysis reveals that elevated CO_2_ mitigates the impact of combined heat wave and drought stress in *Arabidopsis thaliana* at multiple organizational levels. Glob Chang Biol.

[20] Zinta G, AbdElgawad H, Peshev D, Weedon JT, Van den Ende W, Nijs I (2018). Dynamics of metabolic responses to periods of combined heat and drought in *Arabidopsis thaliana* under ambient and elevated atmospheric CO_2_. J Exp Bot.

[21] Fitzgerald GJ, Tausz M, O'Leary G, Mollah MR, Tausz-Posch S, Seneweera S (2016). Elevated atmospheric [CO_2_] can dramatically increase wheat yields in semi-arid environments and buffer against heat waves. Glob Chang Biol.

[22] de Oliveira ED, Bramley H, Siddique KH, Henty S, Berger J, Palta JA (2013). Can elevated CO_2_ combined with high temperature ameliorate the effect of terminal drought in wheat?. Funct Plant Biol.

[23] Dikšaitytė A, Viršilė A, Žaltauskaitė J, Januškaitienė I, Juozapaitienė G (2019). Growth and photosynthetic responses in Brassica napus differ during stress and recovery periods when exposed to combined heat, drought and elevated CO_2_. Plant Physiol Biochem.

[24] Roy J, Picon-Cochard C, Augusti A, Benot ML, Thiery L, Darsonville O (2016). Elevated CO2 maintains grassland net carbon uptake under a future heat and drought extreme. Proc Natl Acad Sci.

[25] Mamatha H, Rao NKS, Laxman RH, Shivashankara KS, Bhatt RM, Pavithra KC (2014). Impact of elevated CO_2_, on growth, physiology, yield, and quality of tomato (*Lycopersicon esculentum* mill) cv. Arka Ashish. Photosynthetica.

[26] Peet MM, Pharr DM, Nelson PV (1991). CO_2_-enhanced yield and foliar deformation among tomato genotypes in elevated CO_2_ environments. Plant Physiol.

[27] Wei Z, Du T, Li X, Fang L, Liu F (2018). Interactive effects of elevated CO2 and N fertilization on yield and quality of tomato grown under reduced irrigation regimes. Front Plant Sci.

[28] Zandalinas SI, Fritschi FB, Mittler R (2020). Signal transduction networks during stress combination. J Exp Bot.

[29] Feng GQ, Li Y, Cheng ZM (2014). Plant molecular and genomic responses to stresses in projected future CO_2_ environment. Crit Rev Plant Sci.

[30] Zhou R, Kong L, Wu Z, Rosenqvist E, Wang Y, Zhao L (2019). Physiological response of tomatoes at drought, heat and their combination followed by recovery. Physiol Plant.

[31] Von Caemmerer SV, Farquhar GD (1981). Some relationships between the biochemistry of photosynthesis and the gas exchange of leaves. Planta.

[32] Ainsworth EA, Long SP (2005). What have we learned from 15 years of free-air CO_2_ enrichment (FACE)? A meta-analytic review of the responses of photosynthesis, canopy properties and plant production to rising CO_2_. New Phytol.

[33] Baker NR, Rosenqvist E (2004). Applications of chlorophyll fluorescence can improve crop production strategies: an examination of future possibilities. J Exp Bot.

[34] Kasajima I (2017). Difference in oxidative stress tolerance between rice cultivars estimated with chlorophyll fluorescence analysis. BMC Res Notes.

[35] Li X, Zhang G, Sun B, Zhang S, Zhang Y, Liao Y (2013). Stimulated leaf dark respiration in tomato in an elevated carbon dioxide atmosphere. Sci Rep.

[36] Yan F, Li X, Liu F (2017). ABA signaling and stomatal control in tomato plants exposure to progressive soil drying under ambient and elevated atmospheric CO_2_ concentration. Environ Exp Bot.

[37] Heath J, Kerstiens G (1997). Effects of elevated CO_2_ on leaf gas exchange in beech and oak at two levels of nutrient supply: consequences for sensitivity to drought in beech. Plant Cell Environ.

[38] Robinson MF, Heath J, Mansfield TA (1998). Disturbances in stomatal behaviour caused by air pollutants. J Exp Bot.

[39] Zhou R, Yu X, Kjaer KH, Rosenqvist E, Ottosen CO, Wu Z (2015). Screening and validation of tomato genotypes under heat stress using F_v_/F_m_ to reveal the physiological mechanism of heat tolerance. Environ Exp Bot.

[40] DeCoster J, Claypool H (2004). Data analysis in SPSS (pp.13).

